# Revolutionizing NSCLC Treatment: Immunotherapy Strategies for EGFR‐TKIs Resistance

**DOI:** 10.1111/crj.70037

**Published:** 2024-12-04

**Authors:** Jin Tian, Zhiqi Shi, Lili Zhao, Peng Liu, Xiaojun Sun, Lin Long, Jianhua Zang, Jun Xiao

**Affiliations:** ^1^ Qingdao Hiser Hospital Affiliated of Qingdao University (Qingdao Traditional Chinese Medicine Hospital) Qingdao Shandong China; ^2^ The First Clinical College of Shandong University of Traditional Chinese Medicine Jinan Shandong China

**Keywords:** EGFR‐TKIs resistance, epidermal growth factor receptor tyrosine kinase inhibitor (EGFR‐TKI), immune checkpoint inhibitors, immunotherapy, non‐small cell lung cancer (NSCLC)

## Abstract

Epidermal growth factor receptor tyrosine kinase inhibitors (EGFR‐TKIs) are the standard treatment choice for advanced non‐small cell lung cancer (NSCLC) patients with EGFR mutations. EGFR‐TKIs have made significant progress in the treatment of advanced NSCLC patients, but drug resistance issues still inevitably arise. The mechanism of drug resistance and subsequent treatment has been current research challenge and priority. Immune checkpoint inhibitors (ICIs) are a new choice for late‐stage NSCLC patients without druggable molecular alterations. Currently, several studies have applied ICIs therapy for NSCLC patients with EGFR‐TKIs resistance and explored the potential efficacy of ICIs. This review elaborates on the current status of immunotherapy after EGFR‐TKIs resistance, including ICIs monotherapy, combined with EGFR‐TKIs, chemotherapy, antiangiogenic drugs, and other therapies.

## Graphical abstract

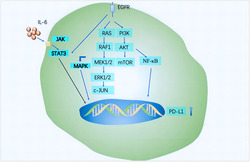

## 1 Introduction

The first‐line treatment for advanced NSCLC is typically divided into targeted therapy, immunotherapy, or chemotherapy/immunotherapy, depending on the driver molecular alterations. One of the most common druggable alterations are mutations in epidermal growth factor receptor (EGFR), which has a mutation rate of up to 51.4% in Asian NSCLC patients [1, 2]. Currently, EGFR tyrosine kinase inhibitors (EGFR‐TKIs) are widely used as the first‐line standard of care for patients with EGFR mutation‐sensitive advanced NSCLC. These targeted therapies have been shown to reduce tumor load, improve patient prognosis, and enhance patient quality of life [3]. The ADAURA study [4] demonstrated the effectiveness of osimertinib, which was the third‐generation EGFR‐TKI drug for patients with T790M mutations after treatment‐resistant first‐ and second‐generation EGFR‐TKIs. It also highlighted the high efficiency of osimertinib in treating central nervous system (CNS) metastatic lesions. However, despite these advancements, the third‐generation osimertinib still encounters resistance, making it crucial to address this issue urgently. ICIs therapy is a well‐known treatment strategy for advanced NSCLC patients without druggable molecular alterations. In recent years, there have been potential studies showing the potency of immunotherapy in NSCLC patients with EGFR‐TKIs resistance. In this article, we will discuss the current status of immunotherapy after EGFR‐TKIs resistance, including ICIs monotherapy, combination therapy with EGFR‐TKIs, chemotherapy, and antiangiogenic drug therapy. The present study aims to provide valuable insights for advanced NSCLC treatments in further research and clinical practice, particularly in the field of patients who are resistant to EGFR‐TKIs.

### 1.1 Upregulation of PD‐L1 Expression Through Signaling Pathways After Resistance to EGFR‐TKIs

Recent findings [5] indicate that PD‐L1, an important immune escape molecule, plays a crucial role in the mechanisms underlying drug resistance in NSCLC. PD‐L1 facilitates the proliferation of NSCLC cells via the Growth Arrest‐Specific 6 (Gas6) C‐Mer proto‐oncogene tyrosine kinase (MerTK) signaling pathway. Additionally, PD‐L1 undergoes nuclear translocation, where it binds to the transcription factor Sp1 and regulates the synthesis and secretion of Gas6 mRNA, thereby further activating the MerTK signaling pathway. It has been demonstrated [5] that Karyopherin β1, also known as KPNB1 serves as a binding partner for intracellular PD‐L1 and enhances its nuclear translocation in NSCLC. Furthermore, nuclear PD‐L1 (nPD‐L1) interacts with Sp1, which transcriptionally regulates Gas6 mRNA synthesis, promotes Gas6 secretion, activates the MerTK signaling pathway, and supports NSCLC proliferation. The activation of MerTK has been shown to promote resistance to EGFR TKIs in NSCLC [6]. The identification of nPD‐L1 through the Gas6/MerTK axis redefines its role in cancer development and offers a new perspective for the development of potential therapeutic strategies for patients who have not responded to PD‐L1 immunotherapy. Due to inevitable drug resistance on EGFR‐TKIs, it is crucial to assess the PD‐L1 expression status and regulatory mechanisms both before and after resistance occurs. A study [7] discovered that PD‐L1 expression significantly increased after resistance to EGFR‐TKIs, thereby facilitating immune evasion by tumors. The expression level of PD‐L1 in the tumor microenvironment (TME) and the tumor mutation burden (TMB) [8] could serve as vital indicators for predicting the response to ICIs in NSCLC patients without EGFR mutation. Additionally, TME‐related indicators such as PD‐L1, TMB, and CD8+ play a crucial role in predicting the response to ICIs in NSCLC patients. Moreover, various factors related to the TME (e.g., tumor‐infiltrating lymphocytes [TIL]) experienced significant changes before and after the development of resistance to EGFR‐TKIs [9]. Previous studies have demonstrated that following resistance to EGFR‐TKIs, the percentage of patients exhibiting high levels of PD‐L1 expression ranged from 14% to 28%. Additionally, the density of CD8 + TIL and FOXp3 + TIL was found to be significantly lower compared with that before EGFR‐TKIs treatment, whereas the expression rate of CD73 + TIL was higher than before treatment [10]. CD73 + TIL has been shown to play a role in mediating tumor immune escape by modulating the TME. On the other hand, CD8 + TIL serves as a predictive marker of low PD‐1 inhibitor efficacy [11], and it is worth noting that high expression of PD‐L1 and CD8 + TIL often coexist. The alterations in the TME induced by EGFR‐TKIs are crucial in determining the effectiveness of subsequent ICIs [12].

**FIGURE 1:**
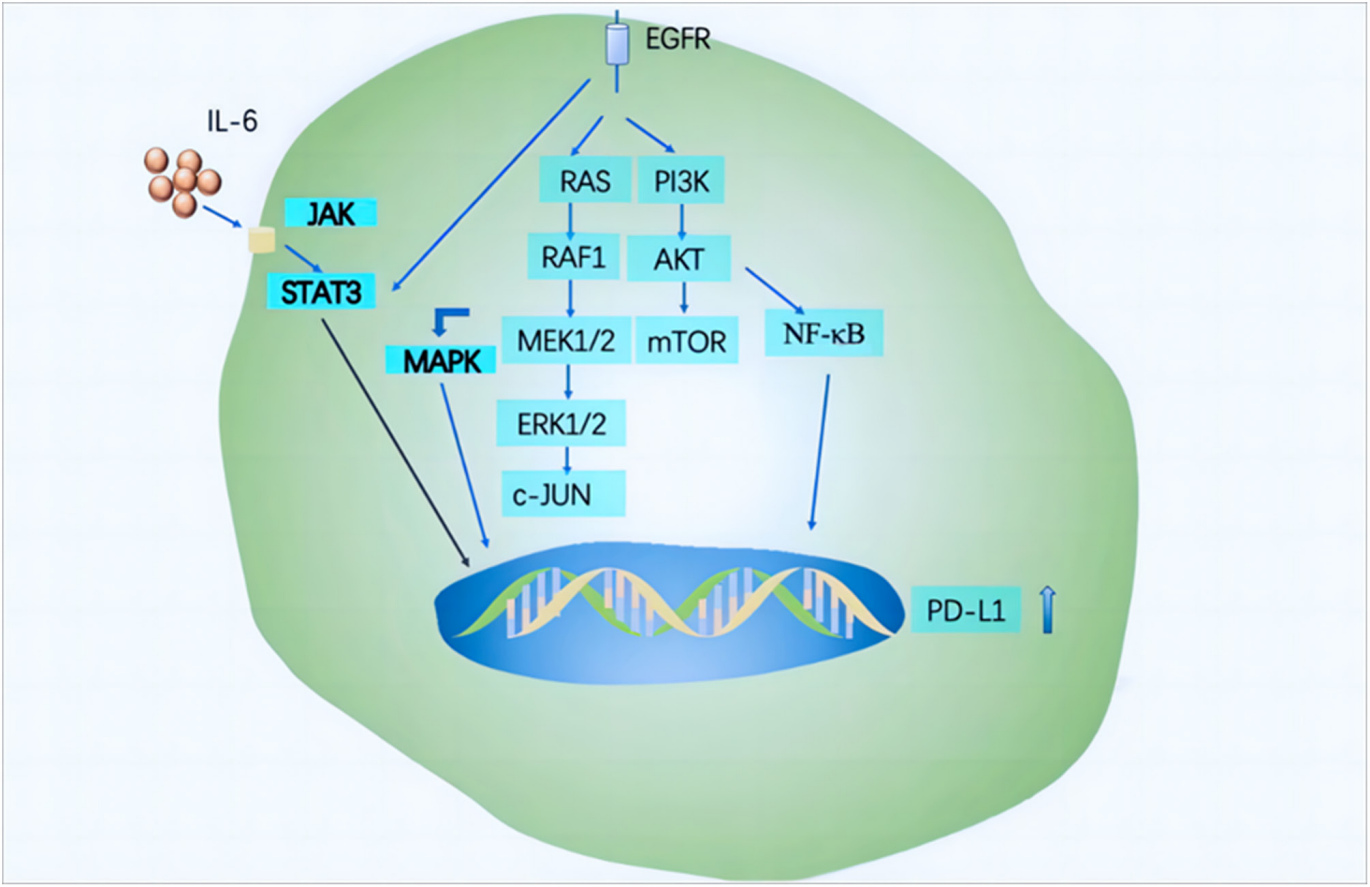
EGFR regulates the expression of PD‐L1.

There are many forms of acquired resistance mechanism in NSCLC patients with EGFR mutations. The resistance mechanism of EGFR‐TKIs involves the PI3K/Akt and MAPK signaling pathways [13]. In NSCLC patients after developing resistance to EGFR‐TKIs, there is a positive correlation between PD‐L1 expression and the mesenchymal‐epithelial transforming factor receptor (c‐MET). One of the mechanisms of EGFR‐TKIs resistance is the binding of hepatocyte growth factor (HGF) ligands and c‐Met, which could activate c‐Met, inducing homodimerization and phosphorylation of intracellular tyrosine residues, eventually promoting tumor immune escape by activating the PI3K/AKT and MAPK signaling pathways and inducing the upregulation of PD‐L1 through activator protein‐1 (AP‐1). Additionally, resistance to EGFR‐TKIs mediated by c‐MET amplification leads to the upregulation of PD‐L1 expression in NSCLC cells through the PI3K/AKT and MAPK signaling pathways. Another factor is the EGFR‐T790M secondary mutation, which upregulates PD‐L1 expression in NSCLC through the PI3K/Akt, MAPK, and NF‐κB signaling pathways. The high expression of PD‐L1 promotes immune escape from tumors. Figure 1 illustrates the mechanism by which EGFR regulates PD‐L1 expression through multiple signaling pathways.

In patients with EGFR L858R mutation who were resistant to gefitinib treatment, it was observed that PD‐L1 expression increased from less than 1% to 50% or more before and after resistance. Additionally, experimental [14] findings revealed that after gefitinib treatment, patients with NSCLC who were resistant to therapy showed elevated expression of NADPH oxidase 4 (NOX4). This increased the expression of NOX4 led to enhanced transcriptional function and accumulation of transcription factor 1 (YY1) in the nucleus. Moreover, NOX4 induced the expression of IL‐8 and PD‐L1 through YY1‐mediated transcriptional activation. By downregulating the expression of YY1/IL‐8/PD‐L1, NOX4 inhibitors can potentially be effective in sensitizing patients who have developed resistance to EGFR‐TKIs to gefitinib once again.

## 2 Exploration of ICIs‐Related Therapeutic Strategies in EGFR‐Mutant NSCLC

### 2.1 ICIs Monotherapy

The Checkmate057 study [15] included 82 patients with EGFR‐mutant non‐squamous NSCLC who had experienced disease progression after treatment with EGFR‐TKIs. The study revealed that NSCLC patients with EGFR‐mutant received second‐line pembrolizumab therapy intend to a better overall survival (OS) than those were treated with docetaxel chemotherapy. It was worth mentioning that this comparison focusing on the EGFR‐mutant NSCLC subgroup and the former achieving better OS via pembrolizumab targeting PD‐1 pathway instead of EGFR directly. Similarly, the Keynote010 study [16] involved 86 patients with NSCLC who had previously received EGFR‐TKIs. It demonstrated that pembrolizumab significantly prolonged OS compared with docetaxel in the EGFR wild‐type group, but no OS benefit was observed in the EGFR‐mutated group. Another subgroup analysis of the OAK study [17] included 85 patients with EGFR mutations and resistance to EGFR‐TKIs who did not experience any benefit from immunotherapy with atezolizumab, as the OS was 10.5 months compared with 16.2 months with docetaxel. All of the aforementioned studies indicate that patients with EGFR mutations do not derive any benefits from immune checkpoint inhibitor (ICI) monotherapy after developing resistance to EGFR‐TKIs therapy. Additionally, the ATLANTIC study included 444 patients who experienced disease progression following at least two previous systemic regimens. The efficacy of durvalumab monotherapy as third‐ or later‐line treatment for advanced NSCLC was evaluated [18]. The patients were divided into three cohorts. Cohort 1 consisted of 111 patients who were EGFR+/ALK+. Within this cohort, patients were further categorized based on their PD‐L1 expression status, with subgroups defined as ≥ 25% and < 25%. Cohort 2 comprised 265 patients who were EGFR−/ALK\−, and their PD‐L1 expression status was also divided into ≥ 25% and < 25% subgroups. Cohort 3 consisted of 68 patients who were EGFR−/ALK− and had a PD‐L1 expression status ≥ 90%. The final OS data remain encouraging across all cohorts, which suggests that durvalumab monotherapy has clinical antitumor activity in patients with advanced NSCLC who have undergone multiple lines of therapy, including EGFR+/ALK+ patients. However, this study did not specifically address the issue of EGFR‐TKI resistance in enrolled patients.

The clinical effect of monotherapy with ICIs after resistance to EGFR‐TKIs is not satisfactory. Wen et al. [19] discovered that the efficacy of ICIs may be influenced by the tumor immune microenvironment, specifically high tumor burden (HTB) and low tumor burden (LTB). Moreover, Yang et al. indicated patients with EGFR mutations intending to a suppressive tumor immune microenvironment [20]. The immune TME can be categorized into immune desert, immune rejected, and immune inflammatory types based on T cell infiltration. Noninflammatory tumors, known as “cold tumors,” belong to the immune desert and immune rejected types, which are insensitive to ICIs. On the other hand, “hot tumors,” or immunoinflammatory types, are heavily infiltrated by T cells [21]. These phenotypes are characterized by the presence of numerous immune cells like CD4+ and CD8+ T cells, as well as pro‐inflammatory cytokines and effectors such as IL‐12, IL‐23, and IL‐2. These factors play a crucial role in the activation and expansion of T cells [22]. Moreover, transforming growth factor‐β (TGF‐β), which was produced by cancer cells and several other cell types presented in the TME, played an important role in immunosuppression. Huang et al. demonstrated that upregulated repression of TGF‐βin NSCLC with EGFR mutations. Besides, high TGF‐β expression inhibited the infiltration and antitumor function of CD8+ T cells, contributing to the “cold” TME of EGFR‐mutated tumors [23]. However, despite these challenges, there is still hope for monotherapy. The Atlantic study [24] revealed that durvalumab extended the median overall survival (mOS) to 16.7 months in patients with EGFR mutations. Moreover, converting “cold tumors” into “hot tumors” and enhancing tumor sensitivity to ICIs is a promising therapeutic strategy. Ongoing studies, such as this one [19], investigate the use of CSF‐1R inhibitor PLX3397 in the early stages of tumor development to increase the effectiveness of ICIs by boosting the number of CD8+ T cells, modifying the TME, and promoting “hot tumor” characteristics.

Research has indicated [25] that the nuclear translocation of PD‐L1 and its acetylation regulation may be critical factors influencing the efficacy of immunotherapy. Specifically, PD‐L1 is acetylated by p300 acetyltransferase at residue K263 within the cytoplasmic structural domain. Conversely, the deacetylation of PD‐L1 by histone deacetylase 2 (HDAC2) facilitates nuclear translocation through interactions with various proteins involved in endocytosis and nuclear import. Furthermore, PD‐L1 deficiency leads to decreased expression of several immune response‐related genes. By genetically or pharmacologically modulating PD‐L1 acetylation to block its nuclear translocation, the expression of immune response‐associated genes can be reprogrammed, thereby enhancing the antitumor response to PD‐1 blockade. Consequently, this study highlights an acetylation‐dependent mechanism regulating PD‐L1 nuclear localization, which in turn governs the expression of immune response genes, suggesting that targeting PD‐L1 translocation could improve the efficacy of PD‐1/PD‐L1 blockade.

### 2.2 Combination Therapy With ICIs

#### 2.2.1 ICIs Combined With EGFR‐TKIs

Immunoglobulin‐like transcript subunit 4 (ILT4), an inhibitory receptor of the immunoglobulin superfamily [26], plays a regulatory role in EGFR‐mutated NSCLC tumor cells by modulating the ERK/Akt signaling pathway. ILT4 promotes the polarization of M2‐type tumor‐associated macrophages (TAMs), leading to T cell dysfunction. Following [27] treatment with EGFR‐TKIs, significant changes were observed in the TME of patients. These changes included an increase in CD8+ T cells, a decrease in forkhead box protein 3 (Foxp3+), regulatory T cells (Tregs cells), and inhibition of M2‐type TAM polarization. These alterations in the TME created a favorable environment for the treatment of ICIs, providing a theoretical basis for the combination of EGFR‐TKIs with ICIs. Furthermore, this study [28] demonstrated that treatment with EGFR‐TKIs (erlotinib) reduced the infiltration of CD4+ regulatory T cells in the TME, resulting in a TME that was conducive to ICIs treatment. The combination of EGFR‐TKIs with a PD‐1 inhibitor showed significant therapeutic benefits and confirmed the antitumor capacity of EGFR‐TKIs, as well as their ability to reverse immunosuppression in the TME. This finding [29] opens up new possibilities for target‐immunity combination therapy.

The feasibility of *ICIs combined with EGFR‐TKIs* is currently being investigated through clinical studies. In the Checkmate012 study [30], the clinical efficacy of nabulizumab in combination with erlotinib for the treatment of patients with advanced NSCLC was evaluated. The median progression‐free survival (mPFS) and mean overall survival (mOS) after treatment with nivolumab in combination with erlotinib were 5.1 months and 18.7 months, respectively. These results confirm the benefits of combination therapy. However, further exploration is needed to identify the populations that could benefit the most from this treatment. Table 1 provides details of relevant clinical trials. It is worth noting that all patients enrolled in the CheckMate 012 study experienced treatment‐related adverse events (TRAE), with five patients experiencing grade 3 TRAE. Further investigation is required to determine if the increase in TRAE is associated with ICIs. The TATTON study [31] was terminated early due to a higher than expected incidence of interstitial lung disease (ILD) in patients receiving osimertinib combined with durvalumab treatment while in the AURA3 study [32], the incidence of ILD with osimertinib monotherapy was only 4%. The consideration of a potential association between the development of ILD and ICIs or combination therapy led to the halt of recruitment in the phase III CAURAL study. In the CAURAL study [33] the objective response rate (ORR) for the osimertinib monotherapy arm and the osimertinib in combination with Durvalumab arm were 80% and 64%, respectively, with no demonstrated benefit from the combination therapy.

**TABLE 1:** Ongoing study of PD‐1/PD‐L1 inhibitors in combination with EGFR‐TKIs in EGFR‐TKIs‐resistant non‐small cell lung cancer.

Trail (NCT identifier)	EGFR‐TKIs	Immune checkpoint inhibitor	*n*	Phase	Status
NCT02364609 (2015)	Afatinib	Pembrolizumab	11	I/I b	Completed
NCT02630186 (2015)	Rociletinib	Atezolizumab	3	I b/II	Terminated
NCT02454933 (2015)	Osimertinib	Durvalumab	344	III	Completed
NCT02143466 (2014)	Osimertinib	Durvalumab/durvalumab + tremelimumab	344	I b	Active, not recruiting
NCT01454102 (2011)	Erlotinib	Nivolumab	472	I	Completed
NCT02039674 (2014)	Erlotinib/Gefitinib	Pembrolizumab	267	I/II	Completed
NCT01998126 (2013)	Erlotinib	Nivolumab/ipilimumab	14	I b	Completed

The clinical efficacy of ICIs combined with EGFR‐TKIs in NSCLC patients after treatment‐resistant EGFR‐TKIs is still a topic of debate. Some studies have shown that these combinations improve the ORR when used as first‐line treatment for NSCLC patients with EGFR mutations. However, there is a concern regarding an increase in immune‐related adverse events (IRAEs). One study [34] reported that the incidence of side effects from immune combination‐targeted therapy was 94.5%, with 47.3% of patients experiencing grade 3 or higher grade side effects. Furthermore, severe IRAEs such as pneumonia and colitis were observed only when osimertinib was combined with ICIs [35] whereas no severe IRAEs occurred when ICIs were combined with the first and second generation EGFR‐TKIs. Interestingly, patients who experienced grade 3 IRAE responses did not have severe IRAEs when they were re‐treated with erlotinib after a 3‐month discontinuation period, suggesting a possible correlation with the short half‐life of osimertinib. The occurrence of IRAEs may be influenced by potential interactions between ICIs and osimertinib. It is recommended to consider delaying the administration of osimertinib or adjusting the order of administration to minimize overlapping drug exposures and mitigate drug–drug interactions, thereby reducing the occurrence of IRAE. Another study [36] found that the risk of ILD increases when nivolumab is combined with EGFR‐TKIs. The proportion of patients who developed ILD was higher (5.09%) when EGFR‐TKIs were combined with nivolumab compared with when EGFR‐TKIs were administered alone (1.22%). Therefore, the incidence of ILD is higher when these drugs are used in combination.

#### 2.2.2 ICIs Combined With Chemotherapy/Antiangiogenic Therapy

Patients with NSCLC who develop resistance to EGFR‐TKIs often have limited treatment options, particularly when it comes to platinum‐containing single‐agent chemotherapy. However, it has been observed that certain chemotherapeutic agents can actually enhance the body's immune response against tumors [37], leading to positive immune effects. These agents can also improve the TME, setting the stage for the combined use of chemotherapy and immunotherapy [38]. In a mouse model, cytotoxic chemotherapy has been found to stimulate pattern recognition receptor (PRR) signaling [39], resulting in immunogenic effects when recognizing endogenous DNA/RNA. This process, known as immunogenic cell death (ICD), activates adaptive immune responses in hosts with an active immune system, thereby boosting the effectiveness of immunotherapy. Furthermore, the combination of chemotherapy and ICIs has shown synergistic effects, including the upregulation of tumor neoantigen presentation mechanisms following chemotherapy‐induced apoptosis. In turn, immunotherapy can enhance a patient's sensitivity to chemotherapy [40].

The Checkmate722 study aimed to compare the effectiveness of atezolizumab combined with chemotherapy versus chemotherapy alone in patients who had previously been treated with first‐ and second‐generation EGFR‐TKIs [36]. The study found a trend towards benefit in patients who received chemotherapy combined with ICIs after resistance to EGFR‐TKIs. The results showed that the median PFS was 5.6 months for the combination therapy group compared with 5.4 months for the chemotherapy alone group, and the median OS was 19.4 months for the combination therapy group compared with 15.9 months for the chemotherapy alone group. However, although there was a trend towards some benefit, no significant difference was observed.

In a retrospective study, the efficacy of PD‐1 inhibitor in NSCLC patients with two common EGFR mutation types, EGFR L858R and EGFR 19del, were analyzed. The study revealed that patients with the EGFR L858R mutation who received chemotherapy combined with immunotherapy had a higher ORR and median PFS compared with patients with the EGFR 19del mutation. These findings were further supported by a retrospective study [41] involving 102 patients who had developed resistance to EGFR‐TKIs and were subsequently treated with ICIs alone or in combination with antiangiogenic agents or chemotherapy (EGFR‐L858R vs. EGFR‐19del mPFS: 6.5 months vs. 3.5 months, *p* = 0.002). The results of this study demonstrated that patients with the EGFR L858R mutation, after developing resistance to EGFR‐TKIs, could benefit from ICIs in combination with chemotherapy or antiangiogenic drugs. Additionally, a correlation was observed between the expression of PD‐L1 and TMB in TME [42, 43, 44]. The immune microenvironment of patients with the L858R mutation exhibited a wider immune distribution and a high expression of CD4 and CD8 in T cells. These findings have important implications for precision therapy, as they facilitate the identification of populations that can benefit from ICIs.

Antiangiogenic drugs play a role in normalizing tumor vasculature, enhancing the infiltration of immune effector cells into the tumor, improving TME, and creating an immune‐supportive environment. This suggests that combining antiangiogenic therapy with immunotherapy can enhance the effectiveness of immunotherapy [45]. Mechanistically, the combination of ICIs with chemotherapy or antiangiogenic drugs exhibits a synergistic mechanism, with the potential to achieve an efficacy greater than the sum of its parts (1 + 1 > 2) [46].

The well‐known Impower150 study [47] aimed to investigate the efficacy and safety of a four‐drug combination regimen consisting of atezolizumab (A), bevacizumab (B), carboplatin (C), and paclitaxel (P) as a first‐line treatment for advanced NSCLC. The study results indicated that patients with EGFR mutations who had previously received EGFR‐TKIs had a mOS of 27.8 months in the ABCP group, which was higher compared with the BCP group (18.1 months). However, no OS benefit was observed in the ACP group (14.9 months). These findings suggest that the four‐drug combination could be a viable therapeutic option for patients who have progressed after EGFR‐TKI treatment. Furthermore, atezolizumab demonstrated promising efficacy in immune combination therapy. The relevant clinical studies are detailed in Tables 2 and 3.

**TABLE 2:** Ongoing studies of PD‐1/PD‐L1 inhibitors plus chemotherapy in EGFR‐tyrosine kinase inhibitor‐resistant non‐small‐cell lung cancer.

Trail (NCT identifier)	Immune checkpoint inhibitor	Chemotherapy	*n*	Phase	Status
NCT03994393 (2023)	Durvalumab/tremelimumab	Cisplatin/carboplatin	100	II	Active
NCT03515837 (2018)/KEYNOTE‐789	Pembrolizumab	Pemetrexed/cisplatin/carboplatin	492	III	Active
NCT04322890 (2020)	PD‐1	Chemotherapy	100		Recruiting
NCT03513666 (2018)	Toripalimab	Pemetrexed + carboplatin	40	II	Active, not recruiting
NCT04405674 (2020)	Tislelizumab	Nab‐paclitaxel + carboplatin/bevacizumab + nab‐paclitaxel	120	II	Recruiting
NCT03802240 (2019)	Sintilimab	Pemetrexed + cisplatin	600	III	Recruiting
NCT04970043 (2021)	Camrelizumab	Pemetrexed + carboplatin	58	II	Not yet recruiting
NCT03924050 (2022)	Toripalimab	Pemetrexed	440	III	Recruiting

**TABLE 3:** Ongoing studies of PD‐1/PD‐L1 inhibitors plus antiangiogenic treatment in EGFR‐tyrosine kinase inhibitor‐resistant non‐small‐cell lung cancer.

Trail (NCT identifier)	Immune checkpoint inhibitor	Antiangiogenic	*n*	Phase	Status
NCT03765775 (2018)	Sintilimab	Anlotinib	20	II	Recruiting
NCT05078931 (2021)	Pembrolizumab	Lenvatinib	35	II	Recruiting
NCT04426825 (2020)	Atezolizumab	Bevacizumab	23	II	Completed
NCT04316351 (2020)	Toripalimab	Anlotinib	60	II	Unknown
NCT04790409 (2021)	Sintilimab	Anlotinib	30	II	Active, not yet recruiting
NCT04517526 (2020)	Platinum‐based chemotherapy + durvalumab	Bevacizumab	60	II	Not recruiting
NCT03647956 (2018)	Atezolizumab	Bevacizumab + carboplatin and pemetrexed	40	IIIB/IV	Unknown
NCT0525827 (2022)	Pembrolizumab	Lenvatinib + pemetrexed + carboplatin	30	II	Recruiting

## 3 Summary and Prospects

Although the use of EGFR‐TKIs has shown benefits for patients with advanced NSCLC and EGFR mutations, it is inevitable that tumor cells will develop drug resistance, thus leading to disease progression. Due to unique immune characteristics in NSCLC with EGFR mutations, the clinical efficacy of ICIs has provided hope for patients with advanced NSCLC. ICI‐based combination therapy has showed inspiring clinical outcomes in NSCLC patients with EGFR mutations after resistance to EGFR‐TKI treatment, especially in patients with the EGFR L858R mutation. Moreover, the combination of immunotherapy plus chemotherapy plus antiangiogenetic agents could be the most effective treatment. However, the optimal ICI‐based strategy for the EGFR‐mutant population remains to be determined. Different subtypes of EGFR mutations, the order of administration, dosage, and timing can also impact the therapeutic effect of ICIs, making it an important area for future research and exploration. Further studies are required to determine the mechanisms underlying the poor response of EGFR‐mutant patients to ICIs. Additionally, understanding the relevant biomarkers of immune‐mediated response is also crucial for identifying the ideal patient population for immunotherapy and achieving precision therapy.

## Author Contributions

J.T. and Z.Q.S. are in charge of literature review, clinical data collection, and validation. L.L.Z., P.L., L.L., and X.J.S. made contributions to manuscript editing. J.H.Z. and J.X. are in charge of manuscript review. All authors read and approved the final manuscript.

## Ethics Statement

The authors have nothing to report.

## Conflicts of Interest

The authors declare no conflicts of interest.

## Data Availability

Data sharing is not applicable to this article as no new data were created or analyzed in this study.
